# Frequency and Associations of Binge-Eating Behavior Patterns With Gastrointestinal Symptoms: A Cross-Sectional Study

**DOI:** 10.7759/cureus.79477

**Published:** 2025-02-22

**Authors:** Isma Haleem, Fatmee Waqar, Ali Akram Khan, Muhammad Umer Shiekh, Ghulam Kazim, Muhammad Rizwan Tariq

**Affiliations:** 1 Community Medicine, King Edward Medical University, Lahore, PAK; 2 Epidemiology and Public Health, King Edward Medical University, Lahore, PAK; 3 Gatroenterology and Hepatology, Mayo Hospital, Lahore, PAK; 4 Surgery, Mayo Hospital, Lahore, PAK; 5 Gastroenterology and Hepatology, Mayo Hospital, Lahore, PAK

**Keywords:** binge eating behavior, binge pattern of eating, frequency of eating, gastrointestinal symptoms, physical symptoms

## Abstract

Background and aim

Binge-eating behavior (BED) poses a significant public health issue worldwide, as it has severe implications for both the physical and gastrointestinal symptoms. The present study evaluated the prevalence of BED with these symptoms. Furthermore, we assessed the correlation between binge eating and gastrointestinal symptoms.

Methods

Two hundred and nineteen patients were enrolled in the study from OPD of the Department of Gastroenterology, Mayo Hospital Lahore. After informed consent, the patient's demographics and complete medical history were obtained. Then, patients were asked about their physical symptoms of heartburn, diarrhea, weight gain, bloating, and pain in the abdomen using a questionnaire. Frequency percentages will be calculated for categorical variables, and chi-square will be used to assess associations.

Results

Out of 219, 114 (52.1%) were male, and 105 (47.9%) were female. The minimum age of the participants was 15, and the maximum age was 56, with a mean age of 31.10 ± 8.214. Eighty-three (27.9%) had one episode of physical gastrointestinal symptoms while having a binge-eating behavioral pattern, 93 (42.5%) had two to three episodes, and 43 (19.6%) had more than three episodes in the last six months. And in these hospital visits, 128 (58.4%) had the symptoms of heartburn. Eighty (36.9%) suffered from weight gain, 86 (39.3%) suffered from constipation/bloating, and 118 (53.9%) suffered from pain in the abdomen, while 86 (39.3%) suffered from diarrhea. There was a significant association between the heartburn and BED pattern, with a p-value of 0.009 (≤0.05), and with diarrhea that was with a p-value of 0.008 (≤0.05).

Conclusion

This study emphasizes the strong correlation between binge-eating habits and gastrointestinal problems, including diarrhea and heartburn. These results highlight the necessity of all-encompassing management approaches that take into account BED's psychological and physical components.

## Introduction

One of the fundamental necessities of life is nutrition. For an organism to grow, develop, and sustain physiological functions, adequate intake of essential nutrients is required, which is obtained by consuming food. However, eating is linked to a number of issues, with eating disorders being among the most prevalent.

Frequent bouts of consuming enormous amounts of food in a short period of time, frequently accompanied by feelings of discomfort and lack of control, are the hallmarks of binge-eating behavior (BEB), a serious eating disorder [1].

Gastrointestinal (GI) symptoms are among the many physical and mental health problems linked to this illness [2]. Children in South Asian nations, such as Bangladesh, Pakistan, and India, have varying prevalence rates of a propensity for eating disorders, ranging from 4% to 38% in non-clinical populations [3]. A pattern of frequent bouts of consuming abnormally large amounts of food in a specific time frame, frequently coupled with a sense of losing control over eating, is known as BEB. In studies, multiple assessment tools are available to assess patients that fall under this category; the Binge Eating Disorder Screener-7 (BEDS-7) scale is one of those. Binge eaters do not purge or over-exercise as a coping mechanism to avoid gaining weight, in contrast to those suffering from other eating disorders like bulimia nervosa. BEB is typified by an inability to regulate one's eating during episodes, which results in misery and subsequent emotions of shame or guilt. It is regarded as a mental illness and is frequently linked to obesity as well as other physical and mental health problems [4].

Developing successful treatment plans requires an understanding of the relationship between BEB patterns and GI symptoms. GI symptoms such as bloating, abdominal pain, constipation, and irritable bowel syndrome (IBS) are frequently more common in those with BEB [5].

These symptoms could be brought on by the psychological stress of binge-eating episodes or the physical effects of overeating, such as altered gut motility or stomach distension [6]. Furthermore, BEB has been connected to elevated inflammation and intestinal permeability, both of which can worsen GI symptoms [7]. Research emphasizes the necessity of a thorough treatment strategy that takes into account GI health as well as dietary habits [8].

Though it also causes physical symptoms, it is regarded as a mental health disorder and is frequently linked to obesity and other physical and psychological health problems. This study aims to evaluate the relationship between BEB and abnormal GI symptoms, including weight gain, diarrhea, heartburn, bloating, and abdominal discomfort, in order to reduce the burden of hospital admissions and enable prompt intervention.

## Materials and methods

Study participants

Two hundred nineteen participants, both male and female, aged 15 to 60 years, had physical gastric symptoms. Patients who were unable to understand the local language, non-cooperative patients (i.e., those who did not give consent), and critical cases were excluded from our study. These volunteers, who had received one or more rounds of health examinations at a Mayo Hospital Lahore outdoor of GI unit between 1st August and 28th October 2024, were the subjects of this cross-sectional study. The trial participants completed a questionnaire about heartburn, diarrhea, weight gain, bloating, and abdominal pain in addition to undergoing a medical examination that included an endoscopy. The study's ultimate participant count was 219 after subjects with endoscopic examinations and biopsy showing malignancy were eliminated. The Institutional Review Board of King Edward Medical University, Lahore, approved the study protocol (IRB no. 452/RC/KEMU). The study was conducted at Mayo Hospital, and all participants provided written informed consent.

Sampling technique

A systematic, self-report questionnaire was completed by each participant, and information was gathered about their medical history as well as socio-demographic characteristics such as sex, age, education, place of residence, marital status, and occupation. 

Nutritional assessment

In one-on-one interviews with all participants, a registered dietitian looked at the individuals' eating patterns. A food frequency questionnaire was used to analyze the frequency and quantity of food intake. The eating pattern was categorized as binge eating.

Statistical analysis

Clinical features, such as diarrhea, weight gain, bloating and abdominal pain, heartburn, and eating episodes, were assessed after recruiting the subjects. The mean and standard deviation were computed for quantitative variables. Frequencies and percentages were computed for qualitative factors such as the frequency of binge-eating episodes, the incidence of physical GI symptoms, hospitalizations for medical reasons, and the level of physical activity. Using chi-square analysis, the association between the binge-eating habit and the physical symptoms of diarrhea, weight gain, bloating, abdominal pain, and heartburn was investigated. p-values below 0.05 are regarded as significant. The statistical software SPSS (IBM SPSS Statistics for Windows, IBM Corp., Version 22.0, Armonk, NY) was used for all analyses. The odds ratio and confidence interval were used to assess how strong the correlation was.

## Results

General characteristics of the subjects

Out of 219 participants, 114 (52.1%) were male, and 105 (47.9%) were female; the minimum age of the participant was 15 years, and the maximum age was 56 years, with a mean age of 31.10 and a standard deviation of 8.214. Sixty-nine (31.5%) participants were married, 106 (48.42%) were unmarried, and 44 (20.1%) were divorced. Regarding the residences, 142 (64.8%) were from urban areas, and the remaining 77 (35.2%) were from rural areas. One hundred six (48.4%) had three or fewer family members, and 113 (51.6%) were from families with more than three members (Table 1).

**Table 1:** Demographic characteristics

Variable	Frequency (n)	Percent (%)	Valid percent (%)	Cumulative percent (%)
Marital status				
Married	69	32	-	
Unmarried	106	48	-	
Divorced	44	20	-	
Residence				
Urban	142	64.8	64.8	64.8
Rural	77	35.2	35.2	100.0
Number of family members				
Three or less	106	48.4	48.4	48.4
More than three	113	51.6	51.6	100.0
Number of episodes of GI symptoms				
One episode	83	37.9	-	
Two to three episodes	93	42.5	-	
More than three episodes	43	19.6	-	

Frequency of gastrointestinal symptoms

Eighty-three (27.9%) had one episode of physical GI symptoms while having binge-eating behavioral patterns. Ninety-three (42.5%) had two to three episodes, and 43(19.6%) had more than three episodes in the last six months. And in these hospital visits, 128 (58.4%) had the symptoms of heartburn. Eighty (36.9%) suffered from weight gain, 86 (39.3%) suffered from constipation/bloating, and n = 118 (53.9%) suffered from abdomen pain, while 86 (39.3%) suffered from diarrhea (Table 2).

**Table 2:** Frequency of gastrointestinal symptoms

Physical gastrointestinal symptoms	Yes	No
Heartburn	128 (58.4%)	91 (41.6%)
Pain abdomen	118 (59.9%)	101 (46.1%)
Diarrhea	86 (39.3%)	133 (60.7%)
Weight gain	80 (36.5%)	139 (63.5%)
Bloating/constipation	86 (39.3%)	133 (60.7%)

The binge-eating behavioral pattern was assessed, a number of episodes of physical GI symptoms of the patients were noted, and there was a significant association seen with the heartburn and BEB pattern, with a p-value of 0.009 (≤0.05) and with diarrhea that was with a p-value of 0.008 (≤0.05). No significant association was seen with other physical symptoms of weight gain with a p-value of 0.074, bloating/constipation with a p-value of 0.057, and pain in the abdomen with a p-value of 0.758 (Table 3).

**Table 3:** Association of binge-eating behavior with physical symptoms

Symptom	Number of episodes	Yes (n)	No (n)	Total (n)	Chi-square value	p-value
Heartburn	One episode	43	40	83	9.535	0.009
	Two to three episodes	51	42	93		
	More than three episodes	34	9	43		
	Total	128	91	219		
Weight gain	One episode	29	54	83	5.208	0.074
	Two to three episodes	29	64	93		
	More than three episodes	22	21	43		
	Total	80	139	219		
Bloating/constipation	One episode	25	58	83	5.741	0.057
	Two to three episodes	39	54	93		
	More than three episodes	22	21	43		
	Total	86	133	219		
Abdomen pain	One episode	46	37	83	0.554	0.758
	Two to three episodes	51	42	93		
	More than three episodes	21	22	43		
	Total	118	101	219		
Diarrhea	One episode	43	40	83	9.771	0.008
	Two to three episodes	32	61	93		
	More than three episodes	11	32	43		
	Total	86	133	219		

## Discussion

Recurrent episodes of excessive food consumption, frequently coupled with a sense of helplessness, are the hallmarks of BED, a behavioral pattern linked to a number of physical and psychological issues. The results of this study demonstrate the frequency and correlation of GI symptoms among those who have engaged in BEBs during the previous six months.

While 42.5% reported two to three episodes, 19.6% reported more than three episodes, and 27.9% reported only one episode of physical GI symptoms during binge-eating episodes, according to our study. Given the stress that these behaviors place on the GI system, these results show that GI symptoms are significantly more common in people with BED. The increased frequency of symptoms with the number of binge-eating episodes underscores the potential cumulative impact of repeated binge eating on physical health.

With 58.4% of people reporting it, heartburn was the most prevalent symptom, followed by weight gain (369.9%), diarrhea (39.3%), bloating or constipation (39.3%), and abdominal pain (53.9%). These symptoms demonstrate the wide variety of GI issues that BEBs can cause. The higher occurrence of heartburn and abdominal pain may be linked to eating large amounts of food in a short time, generally involving high-fat or high-sugar foods that irritate the GI tract. The hallmark of BED is frequent episodes of binge eating, frequently coupled with a feeling of powerlessness. A number of GI ailments have been connected to this habit.

The results showed that binge eating was significantly associated with diarrhea (p = 0.008) and heartburn (p = 0.009) but not with weight gain, bloating/constipation, or abdominal pain.

Current research supports our conclusions on the frequency of GI symptoms in BED patients. These findings are consistent with a 2019 study by Santonicola et al. that patients with eating disorders, particularly BED, often have both upper and lower GI symptoms, highlighting the GI tract's function in controlling food intake [9].

Furthermore, a 2023 study by Murray et al. discovered that people with BED had higher rates of diarrhea and heartburn than people without the disease, which is consistent with our findings that binge-eating practices are significantly associated with these particular GI symptoms [10].

On the other hand, some research has not discovered any meaningful connections between binge-eating habits and specific GI problems. Although GI symptoms are common in people with eating disorders, not all of them are directly linked to binge-eating episodes, according to a 2022 study by Alyami et al. In our study, for example, 53.9% of individuals reported having abdominal pain. However, this did not significantly correlate with binge-eating episodes, according to Alyami et al. [11]. In a similar vein, Camacho-Barcia et al. found that although bloating and constipation are typical symptoms of eating disorders, they may be caused by more than just binge eating; they may also be impacted by dietary composition, stress, or pre-existing GI disorders [12].

A study by Abdulla et al. found that individuals with BEBs reported higher prevalence rates of GI symptoms, including diarrhea. Specifically, 36% of participants engaging in binge eating reported experiencing diarrhea, compared to 22% in those without BEBs. The association remained significant after adjusting for factors such as age, gender, and body mass index [13].

This research by Elhosseiny et al. indicated that individuals with BEBs had a higher prevalence of IBS symptoms, including diarrhea. Approximately 42% of participants with binge eating reported diarrhea-predominant IBS, compared to 18% of those without BEBs. This is consistent with our findings [14].

Contrary to the findings of our study, The study by Dhopatkar et al. examined GI symptoms in patients with various eating disorders, including BED. While it reported a higher prevalence of symptoms like bloating and constipation, the occurrence of diarrhea was not significantly elevated in individuals with BED compared to the general population. Specifically, 15% of BED patients reported diarrhea, similar to 14% in the control group [15].

This resource from the Cleveland Clinic provides an overview of BED, noting that while the disorder is associated with compulsive overeating, it does not always lead to weight gain in every individual. Factors such as metabolism, physical activity, and individual differences play a role in whether binge eating leads to weight gain.

Given the strong correlations seen between binge-eating habits and symptoms like diarrhea and heartburn, medical professionals should closely monitor these symptoms in patients who have been diagnosed with or suspected of having BED. Achieving long-lasting behavioral improvements requires addressing psychological stressors like stress, anxiety, or despair. Smaller, more frequent meals and avoiding trigger foods are two dietary changes that can help lessen GI problems.

The high frequency of GI symptoms among binge eaters highlights the need for early intervention initiatives and more public awareness from a public health perspective. The prevention of BED and its related consequences can be greatly aided by community-based programs that emphasize stress reduction, mental health support, and good eating practices.

Campaigns for education aimed at at-risk groups, such as teenagers and young adults, can help people better grasp the negative effects of binge eating. Early diagnosis and intervention efforts can be improved by teaching medical practitioners to identify the psychological and physical symptoms of BED.

Although this study offers insightful information, it should be noted that it has several limitations. Establishing causal links between binge eating and certain symptoms is limited by the cross-sectional design. The accuracy of reported symptom frequencies may be impacted by recall bias introduced by relying solely on self-reported data.

Longitudinal studies should be taken into account in future studies to have a better understanding of the temporal link between GI symptoms and BEBs. Further understanding of the underlying mechanisms may be obtained by investigating the role that gut microbiota, food composition, and other physiological variables play in mediating these correlations. To determine how well they improve patient outcomes, interventions that address both BEBs and GI symptoms should be reviewed.

## Conclusions

This study emphasizes the strong correlation between binge-eating habits and GI problems, including diarrhea and heartburn. Although they were common, other symptoms like weight gain, bloating/constipation, and abdominal pain did not significantly correlate with bouts of binge eating. These results highlight the necessity of all-encompassing management approaches that take into account BED's psychological and physical components. Affected people's general well-being can be enhanced, and long-term consequences can be avoided with early detection and care.
